# Natalizumab Affects T-Cell Phenotype in Multiple Sclerosis: Implications for JCV Reactivation

**DOI:** 10.1371/journal.pone.0160277

**Published:** 2016-08-03

**Authors:** Marco Iannetta, Maria Antonella Zingaropoli, Anna Bellizzi, Manuela Morreale, Simona Pontecorvo, Alessandra D’Abramo, Alessandra Oliva, Elena Anzivino, Sara Lo Menzo, Claudia D’Agostino, Claudio Maria Mastroianni, Enrico Millefiorini, Valeria Pietropaolo, Ada Francia, Vincenzo Vullo, Maria Rosa Ciardi

**Affiliations:** 1 Department of Public Health and Infectious Diseases, Sapienza University, Rome, Italy; 2 Inserm, U1016, Institut Cochin, Paris, France; 3 Istituto Pasteur-Fondazione Cenci Bolognetti, Rome, Italy; 4 Department of Medical and Surgical Sciences and Biotechnology, Neurovascular Diagnosis Unit, Section of Neurology, Sapienza University, Rome, Italy; 5 Department of Neurology and Psychiatry, Multiple Sclerosis Center, Sapienza University, Rome, Italy; University of Utah, UNITED STATES

## Abstract

The anti-CD49d monoclonal antibody natalizumab is currently an effective therapy against the relapsing-remitting form of multiple sclerosis (RRMS). Natalizumab therapeutic efficacy is limited by the reactivation of the John Cunningham polyomavirus (JCV) and development of progressive multifocal leukoencephalopathy (PML). To correlate natalizumab-induced phenotypic modifications of peripheral blood T-lymphocytes with JCV reactivation, JCV-specific antibodies (serum), JCV-DNA (blood and urine), CD49d expression and relative abundance of peripheral blood T-lymphocyte subsets were longitudinally assessed in 26 natalizumab-treated RRMS patients. Statistical analyses were performed using GraphPad Prism and R. Natalizumab treatment reduced CD49d expression on memory and effector subsets of peripheral blood T-lymphocytes. Moreover, accumulation of peripheral blood CD8^+^ memory and effector cells was observed after 12 and 24 months of treatment. CD4^+^ and CD8^+^ T-lymphocyte immune-activation was increased after 24 months of treatment. Higher percentages of CD8^+^ effectors were observed in subjects with detectable JCV-DNA. Natalizumab reduces CD49d expression on CD8^+^ T-lymphocyte memory and effector subsets, limiting their migration to the central nervous system and determining their accumulation in peripheral blood. Impairment of central nervous system immune surveillance and reactivation of latent JCV, can explain the increased risk of PML development in natalizumab-treated RRMS subjects.

## Introduction

CD49d is an α_4_-integrin that associates with β_1_-integrin to form the very late antigen-4 (VLA-4) or with β_7_-integrin to form the lymphocyte Peyer’s patch adhesion molecule (LPAM). VLA-4 controls the migration of mononuclear leukocytes into the central nervous system (CNS) [1–5]. Blockade of CD49d with the humanized monoclonal antibody natalizumab suppresses trafficking of inflammatory leukocytes into the CNS [3] and leads to a significant decrease in the clinical relapse rate of the relapsing-remitting form of multiple sclerosis (RRMS) [6]. Natalizumab is currently the most effective therapy against RRMS [7], however, its therapeutic efficacy is clouded by reactivation of the John Cunningham polyomavirus (JCV) and development of progressive multifocal leukoencephalopathy (PML) [8].

JCV infects a large portion of individuals worldwide [9, 10] and establishes lifelong persistent infection in kidneys, tonsils, bone marrow and CNS [11–16]. The Stratify JCV® assay is a validated test to measure anti-JCV antibodies in human serum and is currently used to stratify MS patients for higher or lower risk of developing PML [17]. However, due to the occurrence of JCV infection without the development of detectable antibody responses, additional markers are required in order to appropriately stratify patients.

Previous work has shown that natalizumab treatment increases the pool of circulating activated T-cells and other lymphocytes and decreased CD49d surface expression [18]. Therefore, the aim of this study was to longitudinally assess CD49d expression and CD4^+^ and CD8^+^ T-lymphocyte phenotype alterations in the peripheral blood of natalizumab treated RRMS patients, in relationship with JCV reactivation.

## Materials and Methods

### Study population

26 subjects diagnosed with RRMS were enrolled at the Department of Neurology and Psychiatry of the University of Rome “Sapienza” between March 2012 and March 2014. Samples were collected before the first natalizumab infusion (T0), 12 and 24 months post-treatment initiation (T1 and T2, respectively). The therapeutic protocol consisted of administration of 300 mg intravenous natalizumab every 4 weeks. A “washout” period of at least 1 month for immunomodulatory drugs and 6 months for immunosuppressive drugs was mandatory before the initial natalizumab administration. All patients regularly underwent a complete physical and neurological examination and neurological disability was assessed by the Expanded Disability Status Scale (EDSS) score. Sixteen healthy donors (HD), age and sex matched with the RRMS patients, were enrolled as a control group.

This study was approved by the Ethics Committee of Policlinico Umberto I of Rome (protocol number 130/13). All study participants fulfilled the Italian Agency of Drug (AIFA) criteria for natalizumab (Tysabri®) treatment and provided a written informed consent.

### Sample collection

Peripheral blood was collected in 1 ethylenediamine tetra-acetic acid (EDTA) tube, 1 heparin tube and 1 tube without anticoagulants at T0, T1 and T2. Plasma was obtained from EDTA whole blood after centrifugation and stored at -80°C until use. PBMCs were isolated from EDTA whole blood via Ficoll Hypaque (Amersham Biosciences, Uppsala, Sweden) density gradient centrifugation. The number of viable leukocytes was determined by trypan blue exclusion. PBMCs were stored at -80°C until use. Serum was obtained from blood collected without anticoagulants and stored at -80°C until use. Urine was collected in sterile screw-cap containers and stored at -80°C until use. Heparin whole blood was used for multi-color flow cytometry immunophenotyping.

### Detection of JCV antibodies

The presence of JCV specific antibodies in the serum of patients at baseline (n = 16), 12 and 24 months post-natalizumab treatment (n = 26) was assessed using the Stratify JCV^®^ assay, which is a two-step enzyme-linked immunosorbent assay, able to detect anti-JCV specific IgG, with a sensitivity of 350 ng/ml.

### Detection of viral genome by qPCR

DNA was extracted from 1x10^6^ PBMCs, 200μL of EDTA plasma or urine, using the DNeasy® Blood & Tissue Kit (QIAGEN, S.p.A, Milan, Italy), according to the manufacturer’s instructions. DNA yield was determined by measuring the absorbance at 260nm in the eluate. The JCV T-antigen gene was detected and quantified in duplicate in extracted DNA by Real-Time PCR (Q-PCR) using a 7300 Real-Time PCR System (Applied Biosystems, USA), as previously described [19].

Nested PCR was employed for JCV hyper variable non-coding control region (NCCR) analysis, as previously reported [19]. The PCR product was purified with the QIAquick PCR purification kit (QIAGEN, S.p.A., Milan, Italy), and DNA sequencing was performed using an automatic DNA sequencer (Applied Biosystems, model 370 A), according to the manufacturer′s specifications. Sequences were organized and analyzed using the Genetic Computer Group Sequence Analysis software package [19].

### Flow cytometry analysis of CD4^+^ and CD8^+^ T-lymphocytes

**Fig 1:**
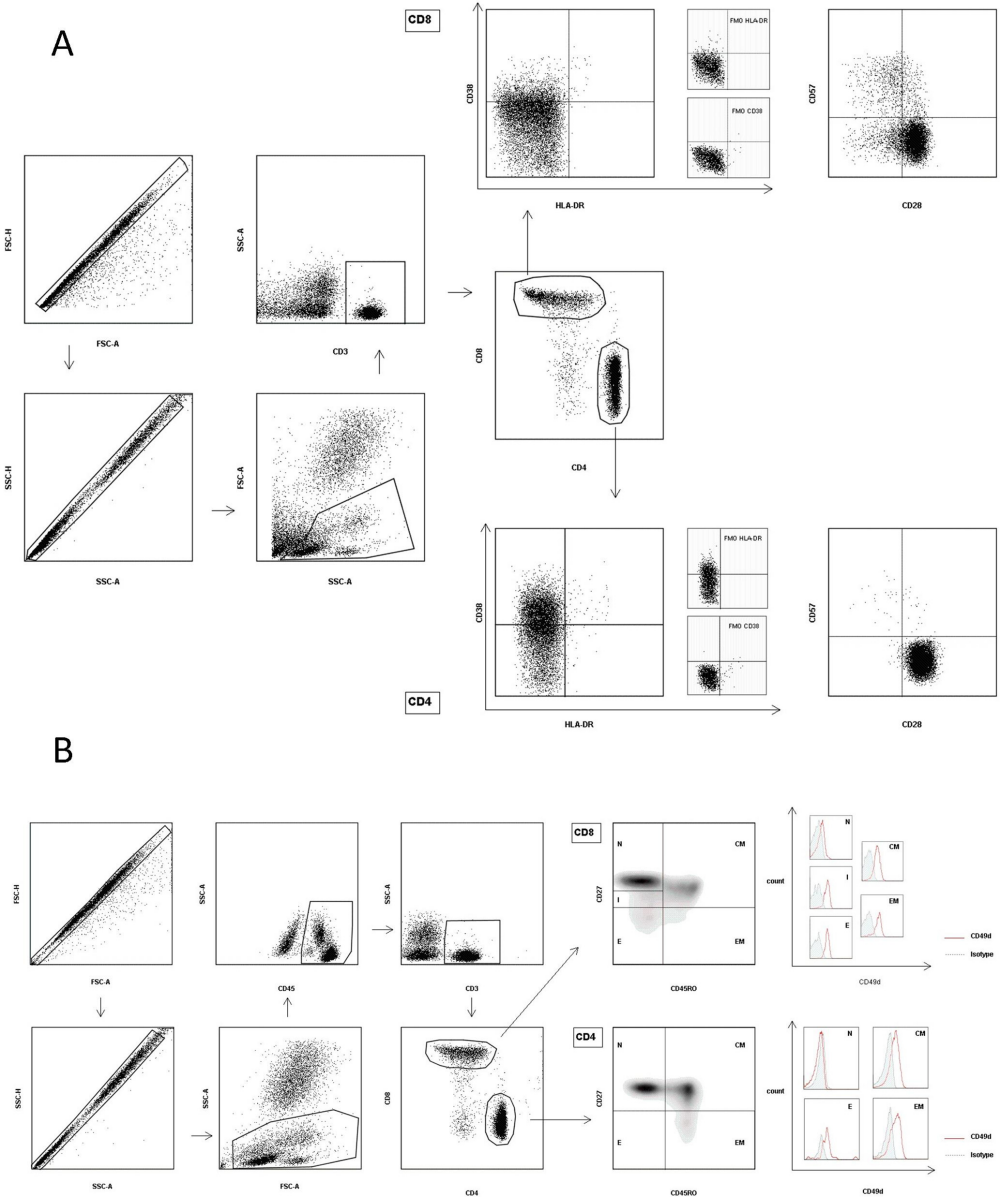
Gating Strategy. (A) After gating for single cells in forward scatter area (FSC-A) versus height (FSC-H) and side scatter area (SSC-A) versus height (SSC-H) plots, lympho-monocytes were identified in a FSC-A SSC-A plot. After gating for CD3^+^ lymphocytes, CD3^+^CD4^+^ and CD3^+^CD8^+^ cells were identified and the percentages of CD38 and HLA-DR double positive events were measured, in order to assess the immune activation of T-cells. Similarly, immune senescence of T-lymphocytes was evaluated by determining the percentage of CD28^-^CD57^+^ events in the CD3^+^CD4^+^ and CD3^+^CD8^+^ gates. Fluorescence minus one (FMO) controls were used to establish HLA-DR and CD38 positive gates. (B) After gating for single cells in forward scatter area (FSC-A) versus height (FSC-H) and side scatter area (SSC-A) versus height (SSC-H) plots, lympho-monocytes were identified in a FSC-A SSC-A plot. After gating for CD45^+^ lympho-monocytes and CD3^+^ T-lymphocytes, CD3^+^CD4^+^ and CD3^+^CD8^+^ maturation subsets were defined as follows: naïve (N, CD45RO^-^ CD27^+^) central memory (CM, CD45RO^+^ CD27^+^), effector memory (EM, CD45RO^+^ CD27^-^) and effector (E, CD45RO^-^ CD27^-^) cells. Only for CD3^+^CD8^+^ T-lymphocytes we identified an intermediate maturation subset (I, CD27^low^CD45RO^-^). The CD49d median fluorescence intensity (MFI) was evaluated in all the CD4^+^ and CD8^+^ maturation subsets. An APC Mouse IgG1 antibody was used as an isotype control.

Immune activation, senescence and maturation subsets of peripheral blood T-lymphocytes were evaluated by flow cytometry using heparin whole blood direct staining. Briefly, 50μl of heparinized blood was incubated with fluorochrome-conjugated monoclonal antibodies for 30 minutes at 4°C in the dark. After red blood cells lysis, cells were washed and analyzed on a MACS Quant flow cytometer (Miltenyi Biotec, Bergisch Gladbach, Germany). The anti-human monoclonal antibodies used were: PacificBlue-CD3 (clone HIT3a), APC/Cy7-CD4 (clone RPA-T4), PE/Cy7-CD8 (clone SK1), FITC-CD28 (clone CD28.2), APC-CD38 (clone HIT2), PacificBlue-CD45 (clone HI30), PE-CD45RO (clone UCHL1), PE-CD57 (clone HCD57) and PerCp/Cy5.5-HLA-DR (clone L243) from Biolegend (Franklin Lakes, New Jersey, USA); PerCp-CD3 (clone SK7), FITC-CD27 (clone M-T271), APC-CD49d (clone 9F10) and APC Mouse IgG1-isotype Control from BD Biosciences (San Jose, California, USA). The anti-CD49d antibody used in these experiments was not impacted by natalizumab binding [20]. CD4 and CD8 Maturation subsets were defined according to CD45RO and CD27 expression as naive (N: CD27^+^CD45RO^-^), central memory (CM: CD27^+^CD45RO^+^), effector memory (EM: CD27^-^CD45RO^+^), effector (E: CD27^-^CD45RO^-^) and (only for CD8) intermediate (I: CD27^low^CD45RO^-^) [21]. The gating strategy is shown in Fig 1.

### Data analyses and statistics

Flow Cytometry data were analyzed using FlowJo Software v. 7.6.5. Statistical analyses were performed using GraphPad Prism version 5 for Windows (GraphPad Software MacKiev). The 2-tailed χ^2^ test or Fisher’s exact test were used for comparing proportions. The Mann-Whitney, Kruskal-Wallis and Friedman tests were used for comparing medians. ROC analysis was performed. Differences were considered significant if p≤0.05. Regression analysis was performed with the statistical software R version 2.15.0 (The R Foundation for Statistical Computing).

## Results

### Description of MS patients

Twenty-six patients (15 females, 11 males) with clinically definite RRMS were included in the study. Sixteen patients were followed for all three time points (T0, T1 and T2), while ten patients were included at T1 and T2.

Sixteen HD, age and sex matched with the 16 patients followed from T0, were enrolled as a control group

Clinical and demographic characteristics are shown in Table 1.

**Table 1:** Demographic and clinical features of RRMS patients.

	Baseline (T0)	12 months (T1)	24 months (T2)	HD
F/M	7/9	15/11	15/11	7/9
Median age in years [IQR]	30.5 [25.2–37]	34 [28.7–40.2]	35 [29.7–41.2]	30 [27.0–34.5]
Median years of disease [IQR]	5.5 [1.25–9.5]	7 [2–12.75]	8 [3–13.75]	N/A
Median EDSS^**a**^ [IQR]	2 [1.25–2.75]	2 [1–3]	2 [1.75–3]	N/A
No therapy^**b**^ (/N)	5/16	8/26	8/26	N/A
Interferon β^**b**^ (/N)	9/16	13/26	13/26	N/A
Mitoxantrone and Interferon β^**b**^ (/N)	1/16	1/26	1/26	N/A
Glatimer acetate^**b**^ (/N)	1/16	4/26	4/26	N/A
Stratify JCV®^**c**^ (+/-)	4/12	11/15	9/17	N/A

F: female; M: male; N: total number of patients; IQR: interquartile range; HD: healthy donors; N/A: not applicable.

a) EDSS: Expanded Disability Status Scale, with values ranging from 0 (normal neurological examination) to 10 (bedridden patient) [Kurtzke, 1983].

b) Therapy before starting natalizumab.

c) Stratify JCV^®^: 2-step virus-like particle-based enzyme-linked immunosorbed assay (ELISA) was performed at T0, T1 and T2, to detect specific anti-JC virus antibodies in serum of the enrolled subjects.

### Stratify JCV^®^

The presence of anti-JCV antibodies was evaluated through the qualitative Stratify JCV^®^ test. In 16 RRMS patients, an increment in the percentage of positivity from T0 to T2 (T0 = 25% [4/16], T1 = 43.7% [7/16], T2 = 37.5% [6/16]) was observed, but the differences were not statistically significant. In all 26 patients, the positivity rate for the stratify test was 42.3% (11/26) at T1 and 34.6% (9/26) at T2, with no significant difference (Table 1).

### JCV PCR and NCCR sequencing

**Fig 2:**
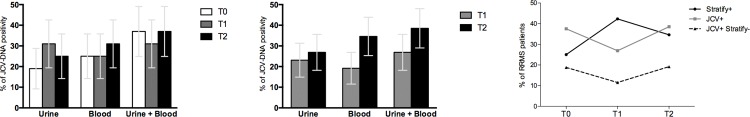
JCV-DNA positivity in urine and blood and discordance with the Stratify JCV® test. (A) Among the 16 patients recruited at all three time points, the percentage of JCV-DNA positivity at T0, T1 and T2 in urine was 18.7% (3/16; standard error [SE] = ±9.8), 31.2% (5/16; SE = ±11.6) and 25% (4/16; SE = ±10.8) respectively, and for blood was 25% (4/16; SE = ±10.8), 25% (4/16; SE = ±10.8) and 31.2% (5/16; SE = ±11.6), respectively. Differences were not statistically significant (urine: p = 0.72, blood: p = 0.90; statistical analysis performed using the 2-tailed χ^2^ test). Combining blood and urine results (positivity corresponds to JCV-DNA detectability in at least one of the two samples), the percentage of positivity was 37.5% (6/16; SE = ±12.1), 31.2% (5/16; SE = ±11.6) and 37.5% (6/16; SE = ±12.1) at T0, T1 and T2, respectively, with no statistically significant differences (p = 0.91; statistical analysis performed using the 2-tailed χ^2^ test). Whiskers represent standard errors. (B) Among all 26 RRMS patients, the percentage of positivity for JCV-DNA at T1 and T2, for urine was 23.1% (6/26; SE = ±8.2) and 26.9% (7/26; SE = ±8.7), and for blood was 19.2% (5/26; SE = ±7.7) and 34.6% (9/26; SE = ±9.3), respectively. Differences were not statistically significant (urine: p = 1.0, p = 0.35; statistical analysis was performed using the 2-tailed Ficher’s exact test). Combining blood and urine JCV-DNA results, the positivity rate was 26.9% (7/26; SE = ±8.7) and 38.5% (10/26; SE = ±9.5) at T1 and T2, respectively, with no statistically significant differences (p = 0.56; statistical analysis was performed using the 2-tailed Ficher’s exact test). Whiskers represent standard errors. (C) In the RRMS patients followed at T0 (n = 16), T1 and T2 (n = 26), the discordance between the PCR for JCV-DNA detection in blood and urine and the Stratify JCV® test was 18,8% (3/16), 11,5% (3/26) and 19,2% (5/26) of cases at T0, T1 and T2, respectively. Stratify+: subjects positive for the Stratify JCV® test. JCV+: subjects with JCV-DNA detection in blood and/or urine. JCV+ Stratify-: subjects positive for JCV-DNA detection in blood and/or urine, and negative for the Stratify JCV® test. RRMS: relapsing-remitting form of multiple sclerosis.

**Fig 3:**
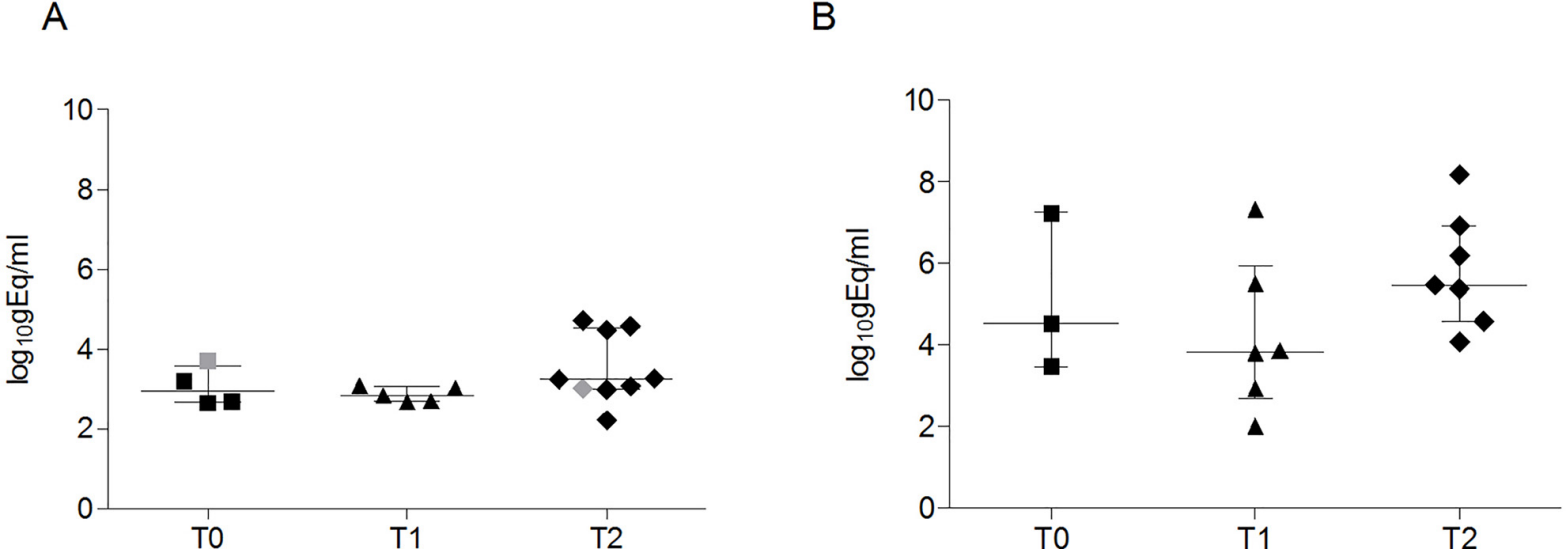
JCV-DNA viral loads. (A) Blood viral load of RRMS patients who resulted positive for JCV-DNA detection at T0 (4/16), T1 (5/26), and T2 (9/26). Symbols in gray represent subjects with JCV-DNA positivity only in PBMC, while plasma resulted negative. No statistical differences were found (one-way ANOVA test for non-parametrical data, Kruskal-Wallis). Results were expressed as log_10_gEq/ml for plasma, and log_10_gEq/10^6^ cells for PBMC. Data are shown as median (lines) and interquartile ranges (whiskers). (B) Urine viral load of the RRMS patients who resulted positive for JCV-DNA detection at T0 (3/16), T1 (6/26), and T2 (7/26). No statistical differences were found (one-way ANOVA test for non-parametrical data, Kruskal-Wallis). Results were expressed as log_10_gEq/ml. Data are shown as median (lines) and interquartile ranges (whiskers).

JCV-DNA detection was performed in blood (plasma or Peripheral blood mononuclear cells [PBMC]s) and urine at T0 for 16 patients and at T1 and T2 for all 26 patients. Subjects were defined as JCV+ when JCV-DNA was detected either in (1) blood, (2) urine or (3) blood and/or urine. Subjects without detectable JCV-DNA in these analyses were defined as JCV-. A non-significant increase of JCV-DNA positivity was found from T0 to T2 in 16 patients (Fig 2A) and from T1 to T2 in all 26 patients (Fig 2B) in either blood, urine or blood and/or urine. No statistical differences between JCV-DNA viral load in blood or urine were detected at any time point (Fig 3). JCV-NCCR sequencing revealed an archetypal organization of this region in all samples. When comparing JCV-DNA and anti-JCV antibody detection as methods for identifying JCV-infected individuals, we found that 3/16 JCV+ subjects (2 in blood and 1 in both blood and urine) at T0, 3/26 JCV+ subjects (1 in blood and 2 in urine) at T1 and 5/26 JCV+ subjects (3 in blood, 2 in both blood and urine) at T2 were negative for JCV serology according to the Stratify JCV® assay (Fig 2C).

### Immunological results

**Fig 4:**
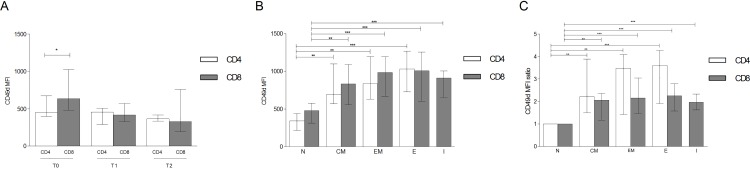
Level of CD49d median fluorescence intensity (MFI) in T-lymphocyte subsets. (A) At baseline (T0, n = 16) CD49d MFI was higher in CD8 (635 [480–1022]) than CD4 (445 [396–673]), p = 0.011. No statistical differences at T1 or T2 (n = 26) were observed. Statistical analysis was performed using the 2-tailed Mann Whitney test. (B) Basal expression of CD49d MFI in CD4^+^ and CD8^+^ N cells (338 [216–435], 474 [310–575], respectively), CM (693 [568–1096]; 830 [554–1087], respectively), EM (834 [631–1195]; 983 [669–1194], respectively), E (1030 [728–1269]; 1006 [603–1256], respectively) and CD8^+^ I (911 [654–1002]). Statistical analysis was performed using a repeated-measures one-way ANOVA test for non-parametrical data (Friedman test). The Dunnett’s post-test was used to compare each CD4^+^ or CD8^+^ subset to the corresponding N subpopulation (n = 16). (C) Fold change in CD49d MFI in T lymphocytes subsets compared to N cells: 2.21 [1.49–3.89] in CD4^+^ CM, 2.06 [1.16–2.36] in CD8^+^ CM, 3.46 [1.41–4.08] in CD4^+^ EM, 2.16 [1.45–3.04] in CD8^+^ EM, 3.60 [1.91–4.27] in CD4^+^ E, 2.25 [1.58–2.79] in CD8^+^ E and 1.96 [1.63–2.33] in CD8^+^ I. Statistical analysis was performed using a repeated-measures one-way ANOVA test for non-parametrical data (Friedman test). The Dunnett’s post-test was used to compare each CD4^+^ or CD8^+^ subset to the corresponding N subpopulation (n = 16). Data are shown as: median [interquartile range]. N: naïve; CM: central memory; EM: effector memory; E: effectors; I: intermediate; *: 0.05<p<0.01; **: 0.01<p<0.001; *** p<0.001.

CD49d expression analysis in peripheral blood T-lymphocytes from all 26 patients revealed statistically higher levels of median fluorescence intensity (MFI) on CD8^+^ compared to CD4^+^ at baseline, whereas no differences at T1 and T2 were observed (Fig 4A). Assessment of maturation subsets within total CD4^+^ and CD8^+^ cells demonstrated lower levels of CD49d MFI in CD4^+^ and CD8^+^ naïve (N) cells compared to central memory (CM), effector memory (EM), effector (E) CD4^+^ and CD8^+^ subsets and the intermediate (I) CD8^+^ subpopulation at T0 (Fig 4B). When assessing fold-change, CD49d expression was two-fold higher in CD8^+^ CM, EM, E, I and CD4^+^ CM compared to the corresponding N subsets, while in CD4^+^ EM and E it was three-fold higher compared to CD4^+^ N cells (Fig 4C). The same expression pattern of CD49d was observed in CD4^+^ and CD8^+^ T-lymphocyte subsets of HD (S1 Fig). Furthermore, no differences in CD49d MFI were found, comparing CD4^+^ and CD8^+^ T-lymphocyte subsets of RRMS patients with those of HD (S2 Fig).

**Fig 5:**
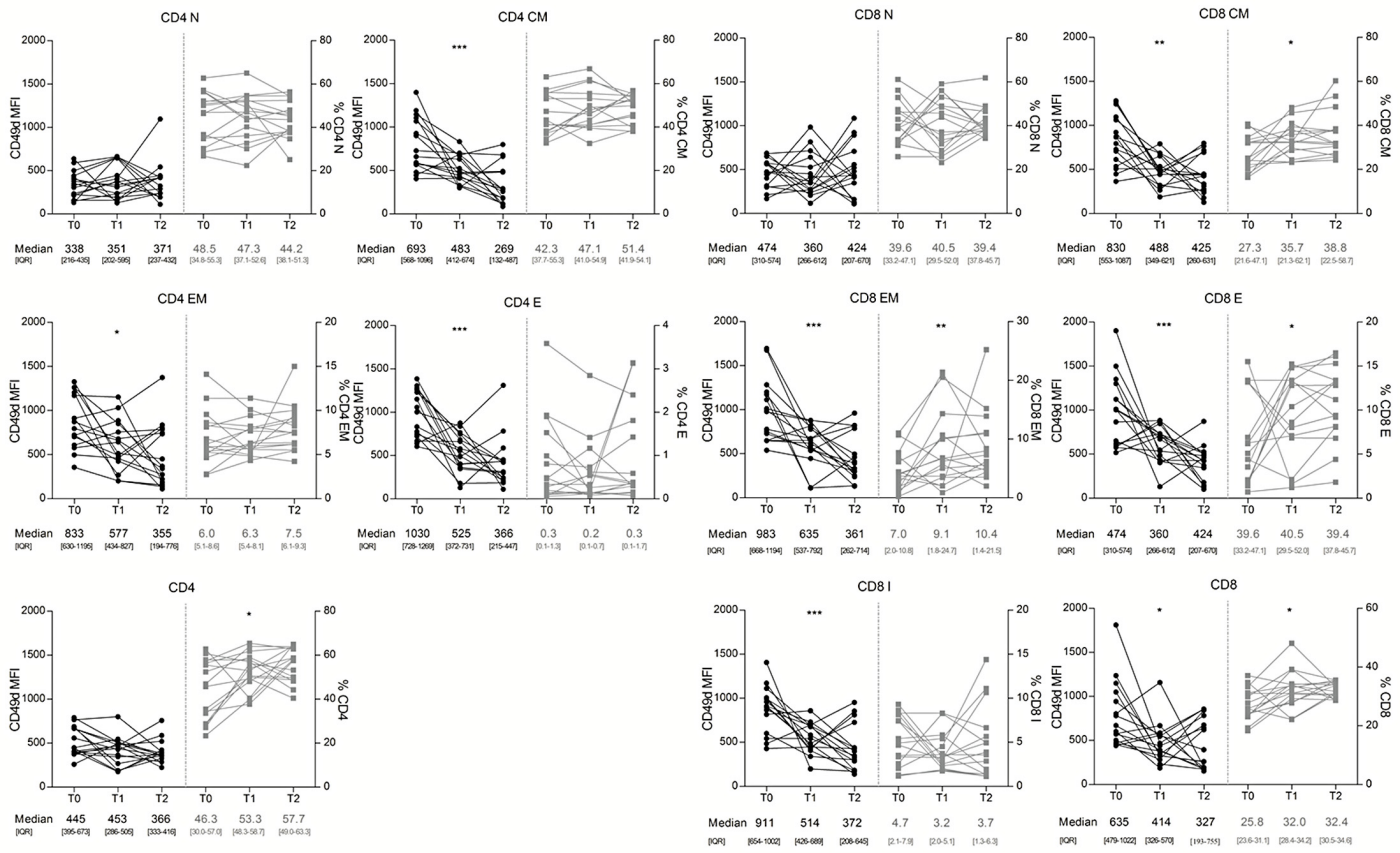
Longitudinal assessment of CD49d median fluorescence intensity (MFI) and T-lymphocyte subsets in 16 RRMS patients. CD49d expression and percentages of CD4^+^ and CD8^+^ N remained constant from T0 to T2. CD49d expression was reduced on CD4^+^ and CD8^+^ CM, EM, E and CD8^+^ I from T0 to T2. The percentage of CD4^+^ CM, EM and E remained constant, while the percentages of CD8^+^ CM, EM and E increased from T0 to T2. CD49d expression on overall CD4^+^ T-lymphocytes remained unchanged, while CD4^+^ percentage was increased over time. CD49d expression was decreased on overall CD8^+^ T lymphocytes and CD8^+^ T lymphocyte percentage was increased from T0 to T2. Statistical analysis was performed using a repeated-measures one-way ANOVA test for non-parametrical data (Friedman test) (n = 16). N: naïve; CM: central memory; EM: effector memory; E: effectors; I: intermediate; IQR: interquartile range; *: 0.05<p<0.01; **: 0.01<p<0.001; *** p<0.001.

In the 16 RRMS patients monitored longitudinally from T0 to T2, a progressive reduction of CD49d MFI values on CD4^+^ and CD8^+^ CM, EM, E and I CD8^+^ was observed. However, no differences were seen for N CD4^+^ and CD8^+^ cells (Fig 5). Overall, the CD4^+^ population showed a constant expression of CD49d, likely because naïve cells represent more than a half of CD4^+^ T-lymphocytes. Conversely, CD49d expression was decreased on the global CD8^+^ population (Fig 5). Longitudinally, CD4^+^ and CD8^+^ N percentages remained stable, while CD8^+^ CM, EM, E and total CD8^+^ T-lymphocytes percentages significantly increased. Despite a non-statistically significant increase of CD4^+^ CM, EM and E subsets, total CD4^+^ T-lymphocytes percentage was significantly augmented (Fig 5).

Finally, elevated levels of CD8^+^ and CD4^+^ immune activation after 24 months of natalizumab treatment were observed (for CD8^+^HLA-DR^+^CD38^+^ T0 = 1.59% [Inter Quartile Range (IQR): 1.01–2.15], T2 = 2.96% [IQR: 1.71–6.01], p = 0.016 and for CD4^+^HLA-DR^+^CD38^+^ T0 = 1.4% [IQR 0.92–1.68], T2 = 1.85% [IQR: 1.24–2.24], p = 0.014; 2-tailed Mann-Whitney test). No statistical differences in immune senescence of CD4^+^ and CD8^+^ T-lymphocytes between T0, T1 and T2 were found.

### Relationship between immunological and virological data

**Fig 6:**
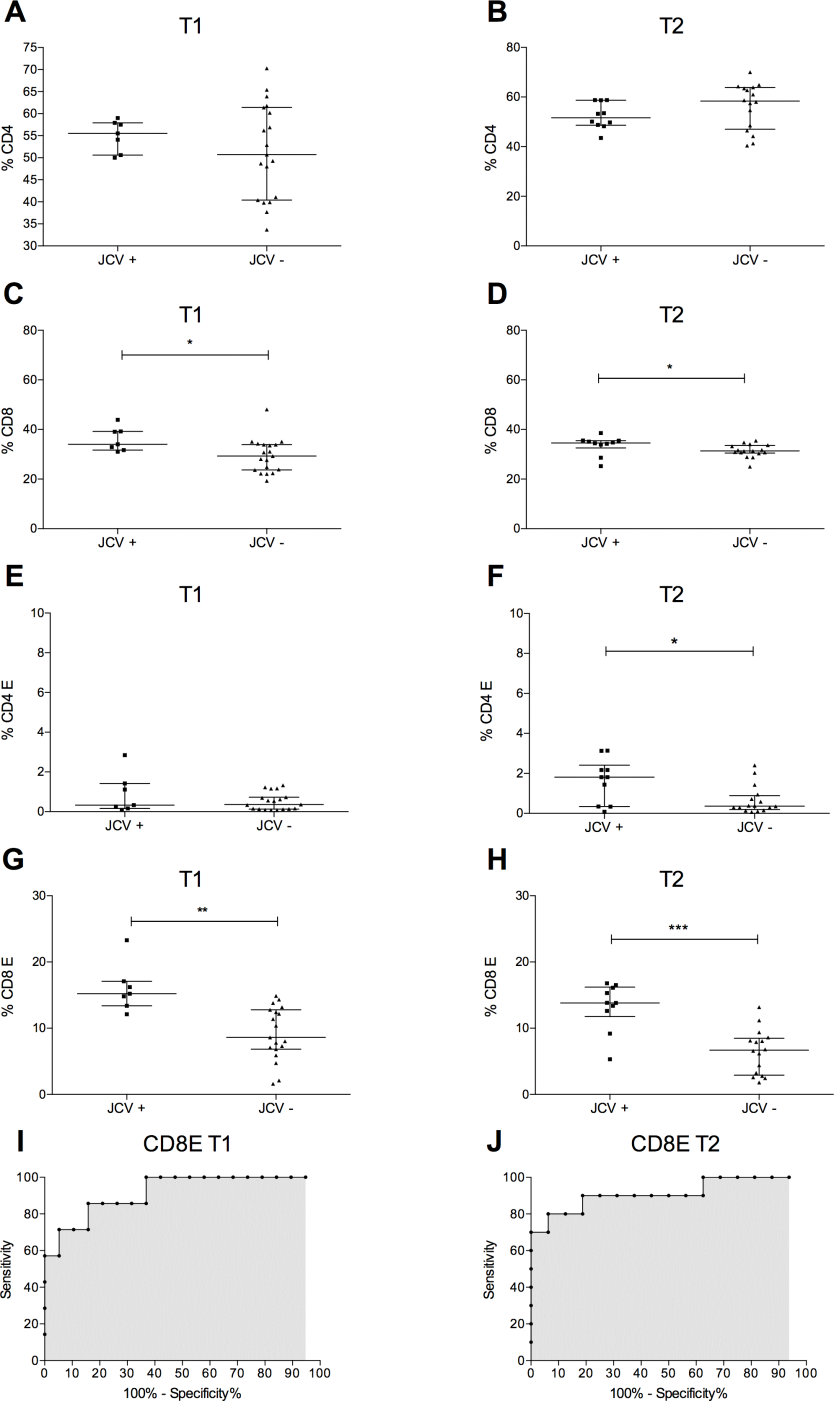
Association of immunological parameters with JCV-DNA positivity. (A-B) The percentages of peripheral blood CD4^+^ T-lymphocyte were unchanged in the JCV+ and JCV- groups at T1 and at T2. Statistical analysis was performed using the 2-tailed Mann Whitney test (n = 26). (C-D) The percentages of peripheral blood CD8^+^ T-lymphocyte were higher in the JCV+ than JCV- group at T1 (34.0% [31.7–39.2] versus 29.3% [23.7–33.9], p = 0.029, respectively) and at T2 (34.6% [32.58–35.53] versus 31.35% [30.50–33.58], p = 0.034, respectively). Statistical analysis was performed using the 2-tailed Mann Whitney test (n = 26). (E-F) The percentage of peripheral blood CD4^+^ E were higher in the JCV+ than JCV- group at T2 (1.81% [0.34–2.4] versus 0.36% [0.19–0.88], p = 0.042, respectively), while no differences were observed at T1 (0.33% [0.09–1.42] versus 0.36% [0.13–0.73], p = 0.53, respectively). Statistical analysis was performed using the 2-tailed Mann Whitney test (n = 26). (G-H) The percentage of peripheral blood CD8^+^ E was higher in the JCV+ than JCV- group at T1 (15.22% [13.4–17.8] versus 8.6% [6.9–12.8], p = 0.0015, respectively) and at T2 (13.82% [11.75–16.2] versus 6.7% [2.9–8.48], p = 0.0006, respectively). Statistical analysis was performed using the 2-tailed Mann Whitney test (n = 26). (I-J) At T1, the ROC curve area for CD8^+^ E was 0.92 with a p = 0.0013 and the cut-off, >13.30, had a sensitivity of 85.71% (CI: 42.13% to 99.64%) and a specificity of 84.21% (CI: 60.42% to 96.62%). At T2 the ROC curve area for CD8^+^ E was 0.91 with a p<0.001 and the cut-off, >11.90, had a sensitivity of 80.00% (CI: 44.39% to 97.48%) and a specificity of 93.75% (CI: 69.77% to 99.84%) (n = 26). Data are shown as: median [interquartile range]. JCV+: subjects with JCV-DNA detection in blood and/or urine. JCV-: subjects negative for JCV-DNA detection in both blood and urine.

To merge immunological and virological data, patients were divided into two groups according to JCV-DNA presence (JCV+) or absence (JCV-) in blood (plasma and or PBMCs) and/or urine samples. A statistically significant increase of total peripheral blood CD8^+^ T-lymphocyte percentages was observed in the JCV+ group compared to JCV- group at T1 and T2 (Fig 6A and 6B); no differences were observed for total CD4^+^ T-lymphocyte frequencies at each follow-up time point. However, peripheral blood CD4^+^ E and CD8^+^ E percentages were higher in the JCV+ than JCV- group, at T2 while only CD8^+^ E were augmented in JCV+ subjects, at T1 (Fig 6C–6F).

Finally, significantly higher levels of CD4 immune activation were only found at T2 in JCV+ compared to JCV- subjects (CD4^+^HLA-DR^+^CD38^+^: 2.12% [IQR: 1.94–2.59] versus 1.58% [IQR: 1.24–2.11], p = 0.0286; 2-tailed Mann-Whitney test).

A logistic regression analysis was used to establish the association of these immunological parameters with the JCV-DNA positivity in natalizumab treated RRMS patients. At T1, only CD8^+^ E percentages were found to be statistically associated to JCV-DNA positivity (p = 0.044), with an odds ratio of 2.39 [95% confidence interval (CI): 1.02–5.59]. At T2 both CD4^+^ E and CD8^+^ E percentages were associated with the presence of JCV-DNA in a simple logistic regression model (for CD4^+^ E the odds ratio was 3.26 [95% CI: 1.18–9.01], p = 0.02. For CD8^+^ E the odds ratio was 1.65 [95% CI: 1.16–2.35], p = 0.01). By using a multiple logistic regression analysis, only CD8^+^ E percentages were statistically associated with the presence of JCV-DNA (p = 0.011), with an odds ratio of 1.68 [95% CI: 1.13–2.50]. Given the association of CD8^+^ E percentage with JCV-DNA positivity in blood and/or urine after 12 and 24 months of natalizumab treatment, the Receiving Operator Curve (ROC) was calculated and was found that a CD8^+^ E percentage greater than 13.30% at T1 and 11.90% at T2 was predictive of JCV-DNA positivity (Fig 6G and 6H).

## Discussion

Although natalizumab is a highly effective drug for RRMS, potential life-threatening side effects, such as PML, are still a major challenge for affected individuals. To date, PML risk assessment and monitoring is based on clinical data, MRI scanning and JCV-specific antibody detection. Indeed, the routine monitoring of JCV serostatus using the Stratify JCV® is now standard of care for RRMS patients [16]. However, due to high JCV seroprevalence both in the general population (60%) and MS patients (51%), the rare occurrence of PML (3.72 per 1000 natalizumab MS patients) makes this tool insufficient in defining the disease development risk [22, 23] underlining the need for appropriate biomarkers to better predict PML development.

The validity of JCV-DNA detection in urine and blood samples as an early marker of PML development is still debated [24–27]. The longitudinal monitoring of JCV-DNA in our cohort showed the occurrence of JCV viruria and transient viremia and the substantial stability of JCV-DNA detection rate after 12 and 24 months of natalizumab treatment, in agreement with previous studies [26, 27]. Interestingly, some patients who were Stratify JCV^®^ negative, had detectable JCV-DNA viruria and/or viremia (Fig 2). Despite an active replication of JCV, these patients are considered at low risk of developing PML, according to the negative result of the Stratify JCV^®^ test. These data indicate that the Stratify JCV® assay may not be as robust as other methods for identifying infected individuals. Furthermore, JCV serostatus alone does not allow for adequate screening of subjects harboring JCV and may therefore be at risk of PML when treated with natalizumab. Although we cannot state that JCV-DNA viruria and/or viremia itself is an early marker of PML, as none of the patients enrolled in this study developed PML, urine and/or blood JCV-DNA detection could be regarded as evidence of JCV presence in latency sites with potential spread to the CNS [28].

Besides JCV-DNA qPCR, NCCR sequencing could help in assessing PML development risk [19]. Recently, deep sequencing techniques of JCV-NCCR in blood and cerebrospinal fluid revealed a close relationship between the quasi-species harbored in these two body compartments [28]. As a consequence, testing for NCCR sequence in blood, an easily accessible body fluid, could provide hints about the viral presence in the CNS [29]. NCCR sequencing performed in all JCV-DNA positive urine and blood samples of our cohort revealed an archetype-like arrangement, which classically is not associated with PML development. This evidence could explain why PML development in these individuals was not observed.

It is known that the rearrangement of JCV-NCCR is necessary for PML development and possibly occurs in a replication-driven sequential series of events [30]. Under this assumption, natalizumab treatment and JCV replication could affect the host immune system, producing changes easily detected using peripheral blood flow cytometric analysis, thus providing viable biomarkers to identify patients at risk for PML development. CD49d monitoring on PBMCs has previously been proposed as a biological marker of natalizumab treatment efficacy being able to identify non-responding patients due to the development of neutralizing antibodies [20].

Several studies have emphasized the role of CD49d in adhesion mechanisms of blood leukocytes to the extracellular matrix and migration from the bloodstream towards tissues and inflammation sites [2–6]. Furthermore, it has been shown that CD49d is a co-stimulatory molecule involved in T-lymphocyte activation, enhancing proliferation in response to OKT3 (an anti-CD3 monoclonal antibody) [31]. Our results demonstrated significantly higher levels of CD49d expression in CD8^+^ compared to CD4^+^ T-lymphocytes at baseline both in RRMS patients and HD; this significant difference was not seen after 12 and 24 months of natalizumab treatment in RRMS patients. Furthermore, we found that in natalizumab untreated MS subjects, CD49d was up-regulated in both CD4^+^ and CD8^+^ T-lymphocytes naïve cells once a memory or effector phenotype was acquired. In particular, for CD8^+^ T-lymphocytes, CD49d was up-regulated in the early stage of differentiation, as demonstrated by the MFI increment in CD8^+^ I subset (Fig 4). The same findings were observed in HD, indicating that CD49d expression pattern follows the same rules in T-lymphocytes of both untreated RRMS patients and HD.

Natalizumab therapy progressively reduced CD49d expression in CD4^+^ and CD8^+^ CM, EM, E and CD8^+^ I cells (Fig 5), leading to the accumulation of CD8^+^ and CD4^+^ T-lymphocytes in peripheral blood. Interestingly, CD8^+^ CM, EM and E exhibited the highest increases. Considering that the increment of these subsets in peripheral blood could correspond to a specular decrease in the CNS, these data indicate that the immune surveillance mechanisms of this compartment could be impaired in natalizumab-treated individuals [31].

The observed higher percentage of CD4^+^ and CD8^+^ HLA-DR^+^CD38^+^ at T2 confirmed the increased activation of T lymphocyte induced by natalizumab treatment. Moreover increased levels of immune activated CD4^+^ HLA-DR^+^CD38^+^ were found in JCV+ subjects, suggesting a direct role of the polyomavirus replication.

Taken together, our data suggest that monitoring of CD49d expression could provide useful information to understand JCV reactivation mechanisms in natalizumab-treated RRMS patients. For that purpose, we directly compared our immunological and virological data and found that higher percentages of peripheral blood CD4^+^ and CD8^+^ E significantly associated with JCV-DNA viremia and/or viruria after 12 and 24 months of natalizumab treatment. Performing logistic regression and ROC analyses, peripheral blood CD8^+^ E percentage was predictive of JCV-DNA positivity (Fig 6). The association between JCV reactivation and natalizumab treatment could be an indirect consequence of CD49d down-regulation. In fact, it has been reported that the number of circulating CD34^+^ hematopoietic stem cells are markedly increased in MS patients during natalizumab treatment [32]. It is not clear if this is a consequence of reduced CD34^+^ precursor adhesion or proliferation due to natalizumab binding to CD49d [33]. Bone marrow CD34^+^ hematopoietic precursors are considered one of the JCV latency sites and their increased circulation could spread the virus through the bloodstream into the CNS [34]. Therefore, the increased JCV circulation, together with the defective CNS immune surveillance, could explain JCV reactivation and PML development in natalizumab treated RRMS patients [35].

Our study has limitations. We did not explore natalizumab-induced modifications of T-lymphocyte phenotype in the CNS, because cerebrospinal fluid samples were not available from any of the patients. In addition, we did not evaluate the JCV-specificity of the memory and effector compartments of T-lymphocytes.

In conclusion, natalizumab treatment decreases CD49d expression on CD4^+^ and CD8^+^ T-lymphocyte memory and effector subsets over time, without affecting naïve cells. As a cansequence of CD49d decreased expression, we observed a progressive accumulation of CD8 memory and effector subsets in peripheral blood. Moreover, natalizumab significatively increased CD4^+^ and CD8^+^ T-lymphocyte immune activation after 24 months of treatment. In our study, increased peripheral blood CD8^+^ E percentages were associated with JCV-DNA positivity. Taken together, these data indicate that natalizumab could mobilize and activate JCV infected cells from latency sites and reduce CD8 effector and memory subsets migration into CNS.

JCV systemic spread from latency sites and impaired immune surveillance mechanisms in the CNS could explain the increased risk of PML development in natalizumab treated RRMS patients.

## Supporting Information

S1 Fig Level of CD49d median fluorescence intensity (MFI) in T-lymphocyte subsets of healthy donors. (A) CD49d MFI in CD4^+^ and CD8^+^ N cells (308 [294–368], 576 [520–605], respectively), CM (722 [571–825]; 901 [614–1097], respectively), EM (855 [691–1028]; 987 [581–1170], respectively), E (850 [461–1070]; 915 [504–1058], respectively) and CD8^+^ I (1007 [780–1066]). Statistical analysis was performed using a repeated-measures one-way ANOVA test for non-parametrical data (Friedman test). The Dunnett’s post-test was used to compare each CD4^+^ or CD8^+^ subset to the corresponding N subpopulation (n = 16). (B) Fold change in CD49d MFI in T lymphocytes subsets compared to N cells: 2.34 [1.49–2.68] in CD4^+^ CM, 1.50 [1.34–1.91] in CD8^+^ CM, 2.59 [2.01–3.25] in CD4^+^ EM, 1.80 [1.15–1.99] in CD8^+^ EM, 2.28 [1.37–3.69] in CD4^+^ E, 1.64 [0.79–1.85] in CD8^+^ E and 1.72 [1.46–1.87] in CD8^+^ I. Statistical analysis was performed using a repeated-measures one-way ANOVA test for non-parametrical data (Friedman test). The Dunnett’s post-test was used to compare each CD4^+^ or CD8^+^ subset to the corresponding N subpopulation (n = 16). Data are shown as: median [interquartile range]. HD: healthy donors; N: naïve; CM: central memory; EM: effector memory; E: effectors; I: intermediate; *: 0.05<p<0.01; **: 0.01<p<0.001; *** p<0.001; ns: not significant.
(TIFF)

S2 Fig Comparison of CD49d MFI in T-lymphocyte of RRMS patients and healthy donors. (A) Comparison of CD49d MFI in CD4 subpopulations of RRMS patients (gray bars, n = 16) and healthy donors (white bars, n = 16). No statistical differences were found. (B) Comparison of CD49d MFI in CD8 subpopulations of RRMS patients (gray bars, n = 16) and healthy donors (white bars, n = 16). No statistical differences were found. Statistical analysis was performed using a non-parametric test (Mann-Whitney). Bars represent median and whiskers represent the interquartile range. HD: healthy donors; N: naïve; CM: central memory; EM: effector memory; E: effectors; I: intermediate; *: 0.05<p<0.01; **: 0.01<p<0.001; *** p<0.001; ns: not significant.
(TIFF)

S3 Fig Percentages of RRMS patients with discordant and concordant results for Stratify JCV®/JCV-DNA tests. The pie charts represent the percentages of RRMS patients who resulted Stratify JCV® negative and JCV-DNA positive (black, T0 = 18.75%, T1 = 11.50, T2 = 19.20), Stratify JCV® negative and JCV-DNA negative (light gray, T0 = 56.25%, T1 = 46.15, T2 = 46.20), Stratify JCV® positive and JCV-DNA positive (gray, T0 = 18.75, T1 = 15.38, T2 = 26.31), Stratify JCV® positive and JCV-DNA negative (dark gray, T0 = 6.25%, T1 = 26,90, T2 = 15.38,). N = 16 for T0. N = 26 for T1 and T2.
(TIFF)
